# That Epidemic of Experts

**Published:** 1898-02

**Authors:** G. S. West

**Affiliations:** Palestine, Texas


### Correspondence.

### That Epidemic of Experts.

PALESTINE, TEXAS, January 25th, 1898.

*To the Editors of the Texas Medical Journal, Austin, Texas.*

GENTLEMEN:--In this month's issue of the TEXAS MEDICAL JOURNAL there appears an article headed "Epidemic of Experts." In that article I find this statement: "It is amusing to read in the medical journals the *pronunciamento* from the following supposed 'experts.' Some of these gentlemen are entirely unknown to the editors of this journal, notwithstanding he has rather an extensive acquaintance with the profession of the State, made during six years' service as secretary of the State Medical Association and thirteen years as editor and publisher of the JOURNAL:

"GALVESTON, TEXAS, October 16th, 1897.

"WHEREAS, after having heard a full explanation of the prevailing epidemic now in Galveston, and also a thorough explanation of the course and results of all of the suspected cases of yellow fever, as diagnosed by Drs. Guiteras and Swearingen and some of the local physicians, we, the visiting physicians of the interior of Texas, resolve that we do not believe that there is a case of yellow fever in the State of Texas.

"*Resolved*, further, That we do not believe that there has been a case of yellow fever within the State of Texas within the past twelve months. While we believe that Drs. Guiteras and Swearingen, and others who have made diagnoses of yellow fever in Galveston and Houston, were honest and conscientious in their diagnoses, the results have not borne out such diagnosis. (Signed.) J. F. Collier, M. D., Health Officer Montgomery County; J. E. Vann, M. D., Health Officer Trinity County; J. S. Wooters, M. D., Crockett; C. I. Holt, M. D., Kilgore; Lawrence Corley, M. D., Crockett; F. C. Woodard, M. D., Grapeland Board of Health; W. N. Hooper, M. D., Conroe; G. S. West, M. D., Palestine; W. G. Jameson, M. D., Palestine, Chief Surgeon International & Great Northern Railroad; J. L. Hall, Crockett; G. L. Davis, Trinity."

Then you say:

"These gentlemen could with propriety certify that they had not seen a case in Galveston which they believed to be yellow fever; but in the name of consistency and common sense, upon what do they predicate the belief that there has not been a case of yellow fever in Galveston? By what did they judge? On what data did they rely to ascertain this astonishing belief? It is a stultification. This declaration on the part of the physicians of the far interior of the State, in face of the opinion of Dr. Guiteras, an expert of international fame; State Health Officer Swearingen, a yellow fever veteran; Prof. West, ex-Professor Practice of Medicine, Texas Medical College; Prof. J. W. McLaughlin, Professor Practice of Medicine, Texas Medical College; Dr. Truehart of Galveston, who practiced through the epidemic of 1867, and a number of other experienced yellow fever physicians, places the signers in a ridiculous position."

Very well; let us see: In the month of October, 1897, yellow fever was very prevalent in the city of New Orleans. At the same time dengue was epidemic in this State, not only in the sea-board cities, but throughout the State, the epidemic was intensely violent, the symptoms very pronounced, and the symptoms, many of them, very closely resembling that of yellow fever. Dr. Guiteras, fresh from New Orleans, seeing almost identically the same symptoms, widely diffused, pronounced yellow fever to be prevailing in the city of Galveston. Now note the difference in the two diseases. Dengue is an acute febrile disease which prevails as an epidemic; it is characterized by two paroxysms of fever, with an intermission of variable deviation between them. The first paroxysm accompanied by high fever and joint swelling and an eruption. The second subsiding suddenly with some critical evacuation. Usually the onset of the disease is sudden. The patient is taken in full health, often awakens from sleep with intense headache, burning pain in the temple, backache and severe aching of all the joints. With the headache there is also great intolerance of light and sound; the face is flushed and hot; the tongue coated; a good deal of burning pain is felt in the abdomen; there is nausea and vomiting, during which a quantity of bilious matter comes up and scarcely anything is retained; constipation persists, the action of the heart is rapid, the pulse strong and beating at 140 or higher, the duration is variable, lasting from several hours to as many days. It may cease rather suddenly with critical phenomena, or slowly by lysis; this I consider to be the main feature of dengue fever. In this last epidemic there was more jaundice than I have formerly observed.

Yellow fever on the contrary is a pestilential fever of continuous and specific type, and it always presents two well defined stages: the first characterized by intense pain in the head and back; injected eyes; rapid circulation; elevated temperature from 106 to 110 which may extend from twenty-four to one hundred and sixty hours, according to the severity of the disease; the second characterized by depression of the nervous and muscular forces, and of the general and capillary circulation; capillary congestion; slow and intermittent pulse; jaundice; urinary suppression; passive hemorrhages from the stomach and bowels, nose, tongue, gums, uterus, vaginas, gall bladder and ureters, and in extreme cases from the eyes, ears and skin; black vomit; convulsions; delirium and coma.

Now, Dr. Daniel, is it contended for one moment by anybody that there was a single case in the city of Galveston during the whole year of 1897 presenting these symptome? From all the cases combined could an ensemble have been arranged of symptoms necessary to present one typical case of yellow fever? If so, I was most wofully misinformed and deceived. There can be no doubt about yellow fever; it is like typhoid fever, or pneumonia, or any other self-limited disease; cannot be cured in four or five days, any more than they can. Yellow fever is a continued pestilential and self-limited disease, presenting two well defined stages; the first characterized by active chemical changes in the blood and organs, attended with elevation of temperature and aberration of nervous action. And the other a stage of depression, induced both by the sedative action of the febrile poison, and by profound changes excited in the blood, and in certain organs, viz: the heart, liver and kidneys, and by the direct sedative and poisonous action of the excrementitious matter retained in the blood, in consequence of the failure, arrest or perversion of the functions of the liver and kidneys, and by the arrest or perversion of the digestive function, in consequence of the action of the yellow fever poison in causing perverted nervous action, capillary congestion and active desquamation of the secretory cells of the stomach and in consequence of the elimination of the gastric mucous membrane, of certain constituents of blood and urine, viz: urea and carbonate of ammonia. The action of the yellow fever poison is the same in all cases, either mild or severe; the progress and termination of the cases, as well as the manifestation of the various symptoms, depends upon the extent of the action of the poison, the condition of the system at the time of the introduction, the peculiarities of the constitution and the supervention of other diseased states. In these days when there are so many opportunities to investigate disease, and in a city like Galveston, with a medical college, with presumably an accomplished microscopist attached, why was not a microscopical examination made? Is it not known that the blood of yellow fever patients under the microscope differs essentially from that of other diseases? Do you not know, or does the veteran yellow fever State Health Officer know that the blood globules rapidly assume a crenated form with minute transudations upon the surfaces? In some yellow fever patients the blood often contains small particles possessing a vibratory motion, and bacteria often has been observed, and the filaments of a delicate fungus in the blood of yellow fever patients. Blood of yellow fever patients allowed to stand micrococci fungi and bacteria are soon developed, and if permitted to stand four or five hours and injected under the skin of a dog, proves rapidly fatal. Certain constituents of the blood, as the albumen and fibrin are altered physically and chemically, in the early stages of yellow fever, and as the disease advances, from the causes just specified, certain excrementitious matters, which in a state of health are continuously eliminated, accumulate in the circulating fluid, and by their direct action upon the elements of the blood, and upon the nervous system, and by their disturbing action upon the processes of nutrition and digestion, still further alter the physical and chemical and vital properties of the fluid. It is not my intention to write a thesis upon yellow fever, but I desire to mention some of the most well known symptoms and name some of the many tests that might have been made and were not.

Dr. Guiteras seemed to have cast a glamor of hypnotism over all with whom he came in contact, and his word was law. The sleep was short, the awakening sudden and the truth triumphant. It seems to me to be a very serious thing to quarantine a large commercial city like Galveston, to shut it out from the world. There is no way of estimating the damage done, therefore every precaution should be taken.

On the 16th day of last October I was invited by Dr. Jameson to accompany him to Houston and Galveston to see if there was any yellow fever there. I accepted his invitation, and upon arriving in Galveston was introduced to Dr. Fisher and other members of the Board of Health, and I enquired of them what were the symtoms of the cases pronounced to be yellow fever, and how long they remained ill; if any of them died, if so, how many of them died; was any post mortem examination made, if so what was the appearance and condition of the liver, heart, stomach, spleen and kidneys? Their answers were given promptly, freely, and I am certain, truthfully; and from their answers and assurances, I know there has been no yellow fever there. Another fact assured me; if there had been any yellow fever there would still have been some remaining there. Yellow fever does not enter a city and impregnate ten or fifteen cases and leave. When it comes, it comes to stay until heavy frost. The facts above stated are what I predicate by belief upon, and that is why I judged. The want of symptoms confirmatory of the disease yellow fever, the unusual feature of its sudden and complete decline, a feature never before heard of, to my mind made assurance doubly sure.

I have great respect for those in authority, and I am very chary how I bump up against the pet theories of others; but what I know, I know, and no man or set of men, I care not what or how others may esteem them, or however great their fame, can induce me to stulify myself for one moment. I know yellow fever, if I am living in the "far interior," and while I never claimed to be a yellow fever expert, if I should, would I not be entitled to the appellation? I do not know what is considered an expert in yellow fever, but this I do know, that I served through the epidemic in New Orleans, La., in 1853; in Savannah, Ga., in 1854; in Norfork, Va., from August until December, 1855, where out of a population of nine thousand, thirty-five hundred of them were buried, and nine resident physicians, and forty volunteer physicians died, and I do know that I was one of nine volunteer physicians taken down with yellow fever at one time, and that two of them besides myself recovered, the balance died; in 1867, as you know, I served through the epidemic of yellow fever in New Orleans, La., and during the civil war I was sent to Wilmington, N. C. when it was reported there, and again in Bryan, Texas. I have been Health Officer of this city since 1872 whenever an epidemic disease has appeared. Have I any claims as an expert? I have performed more than one hundred post mortem examinations on yellow fever patients. I have treated more cases of yellow fever than Dr. Guiteras, I know, and more than any doctor in Texas has seen. I have practiced my profession more than fifty years; am in my seventy-fifth year, and Dr. Daniel, I am not unknown to you personally, and our acquaintance antedates your knowledge of Texas. I am not given to boasting as you know. I did not write the resolutions, but I signed them because I knew them to be true. I am now as I have always been and hope yet to remain,

Your friend,

G. S. WEST, M. D.

